# Relationship between nutrition knowledge and nutritional adequacy in Japanese university students: a cross-sectional study

**DOI:** 10.1017/jns.2025.5

**Published:** 2025-02-05

**Authors:** Yatsuki Yanagihara, Aiko Narumi-Hyakutake

**Affiliations:** 1 College of Gastronomy Management, Ritsumeikan University, 1-1-1 Nojihigashi, Kusatsu 525-8577, Japan; 2 Faculty of Nutrition, Kobe Gakuin University, 518 Ikawadanicho-Arise, Nishi, Kobe 651-2180, Japan

**Keywords:** diet quality, dietary habits, Japanese adults, nutrition knowledge, nutritional adequacy, NKQ, nutrition knowledge questionnaire, SD, standard deviation, BDHQ, brief-type self-administered diet history questionnaire, DRIs, dietary reference intakes, DG, tentative dietary goal for preventing lifestyle-related diseases, EAR, estimated average requirement, EER, estimated energy requirement, SFA, saturated fatty acid, BMI, body mass index

## Abstract

The aim of this study was to examine the relationship between nutrition knowledge and nutritional adequacy among Japanese university students. This cross-sectional study was conducted in 2018 at a university located in Hyogo Prefecture, Kobe, Japan, and 801 students from various academic disciplines were enrolled. Eligible participants were students aged more than 18 years, encompassing all years of study. Nutrition knowledge data were obtained using a nutrition knowledge questionnaire (NKQ) for Japanese adults. Participants were classified into three nutrition knowledge groups according to their total NKQ scores [mean ± standard deviation; all (64.7 ± 15.4%), low (48.1 ± 13.8%), medium (68.3 ± 2.8%), and high (78.5 ± 4.2%)]. Participants reported their dietary habits in the preceding month using a brief self-administered diet history questionnaire. Differences in nutritional adequacy among groups were determined using logistic regression and covariance analyses, adjusted for potential confounding factors. The adequacy of each nutrient was quantified as the proportion of participants with nutrient intake that fell outside the reference range. Although the intakes of only a few nutrients and foods were associated with nutrition knowledge, the total number of nutrients below the estimated average requirement was lower in the high nutrition knowledge group (3.1 ± 2.7) than in the low nutrition knowledge group (3.6 ± 2.9) (*P* = 0.046). In conclusion, the nutrition knowledge level of Japanese university students is associated with nutritional adequacy but may partially affect eating habits. Future longitudinal studies must clarify the causal and dose-response relationships between nutrition knowledge and dietary habits.

## Introduction

Unhealthy dietary patterns are associated with a multitude of health risks, including susceptibility to numerous noncommunicable diseases, making the cultivation of healthy eating habits a vital aspect within the realm of public health.^(1)^ Additionally, many lifestyle habits, including eating habits, develop from adolescence to young adulthood.^(2)^ Thus, fostering healthy eating habits in young adults is important for future health. However, young adults often exhibit unhealthy dietary habits. A systematic assessment of 187 countries, including many Western and Asian countries, revealed that young adults had lower diet quality scores than other age groups.^(3)^ Specifically, young adults have a decreased intake of fruits, vegetables, and whole grains and increased intake of energy-dense, nutrient-poor foods, such as sugar-sweetened beverages and processed meats.^(3)^ Furthermore, university students, including those in Japan, often face dietary challenges owing to habits such as high rates of skipping breakfast, frequent dining at fast food establishments, low consumption of fruits and vegetables, insufficient intake of minerals, and high consumption of oils and saturated fatty acids.^(4–6)^ Thus, investigating factors that influence the eating habits of young adults and college students, who face greater dietary challenges than other age groups, is important in building their lifelong health.

Nutrition knowledge is recognised as one of the factors influencing dietary behaviour, with global efforts aimed at promoting education to improve nutrition.^(7–9)^ A previous systematic review showed that groups with high nutrition knowledge tend to exhibit higher fruit and vegetable consumption and consume less fat and oil than those with low nutrition knowledge, suggesting a positive link between nutrition knowledge and healthy dietary habits.^(10)^ Nevertheless, limitations have been pointed out, such as bias in demographic characteristics and the use of questionnaires with unconfirmed validity.^(10)^

While reports in Japan on this topic are limited, evidence suggests that elementary school students with higher nutrition knowledge exhibit increased fruit and vegetable intake.^(11)^ Furthermore, a survey among Japanese adults indicated a positive association between overall diet quality and nutrition knowledge.^(12)^ However, nutrition knowledge may vary depending on the demographic characteristics and socioeconomic factors of the individual.^(13)^ Moreover, studies examining the relationship between nutrition knowledge and dietary intake have yielded diverse results, thereby preventing the formulation of definitive conclusions.^(10)^ Additionally, only a few studies have examined the relationship between nutrition knowledge and overall diet quality, with none specifically focusing on young Japanese adults.

Therefore, in this study, we aimed to examine the relationship between nutrition knowledge and adequacy of nutrient intake among Japanese university students. The findings of this study may aid in interpreting the relationship between nutrition knowledge and diet quality in many countries where dietary challenges exist among young adults, such as those in Japan.

## Experimental methods

### Participants

We conducted an anonymous, self-administered paper questionnaire survey targeting students at a university in Hyogo Prefecture, Kobe, Japan, between September and November 2018. The university enrols over 10,000 students in both health-related courses (e.g., nutrition, pharmacy, physical therapy, and occupational therapy) and non-health-related courses (e.g., humanities, law, and business), providing the opportunity to recruit students across a range of academic disciplines. Convenience samples were recruited through study researchers or volunteer collaborators (recruiters). A recruiter attended the collaborators’ classes to present the study and asked students to complete the survey. This recruiter explained the purpose of the survey, outlined the procedure to students taking classes, and distributed questionnaires to every student attending the class on the day of the survey. All participants were invited during their classes in the classroom to participate in the present study and were considered eligible to participate if they provided informed consent for the collection of their data. Eligible participants included students over 18 years of age from all years of study. Students who had received dietary counselling from a doctor or dietitian and women who were pregnant or lactating were excluded from the study.

### Materials

In this study, we used three questionnaires, that is, a lifestyle questionnaire, a nutrition knowledge questionnaire for adults (NKQ for adults), and a brief-type self-administered diet history questionnaire (BDHQ).

### Nutrition knowledge

The NKQ for adults was developed by Parmenter and Wardle^(14)^ and reorganised for the Japanese population by Asakura *et al.* ^(11)^ The questionnaire consisted of 84 questions, divided into four sections: 1) knowledge about foods as nutrient sources, 2) physiological functions of nutrients in the human body, 3) awareness of dietary recommendations, and 4) relationship between nutrients and health outcomes. The percentage of correct answers for each section and the total were calculated in the NKQ for adults. The internal consistency between sections in the questionnaires was evaluated using Cronbach’s alpha; the method for calculating Cronbach’s alpha has been explained previously.^(11)^ Cronbach’s alpha for the present study was 0.68, whereas that for the previous study was 0.59. As the exclusion of the section on awareness of dietary recommendations improved Cronbach’s alpha of the questionnaire, this section (5 questions) was not included in the subsequent analysis as in previous studies (Cronbach’s alpha: previous study,^(11)^ 0.69; present study, 0.76). Finally, the percentage of correct answers to the 79 questions was defined as the NKQ score. The participants were divided into three levels (tertiles) of the nutrition knowledge groups according to NKQ scores (min–max: low, 0–62.0%; medium, 63.3–72.2%; high, 73.4–91.1%).

### Dietary assessment

Dietary habits in the preceding month were assessed using the BDHQ. Details regarding the structure, method for calculating dietary intake, and validity of commonly studied food and nutrient intakes using the BDHQ have been published previously.^(15,16)^ The BDHQ is a four-page 95-question item self-administered questionnaire consisting of structured questions about the consumption frequency of selected foods commonly consumed in Japan as well as general dietary behaviour and usual cooking methods. Estimates of the daily intake of foods (58 items in total), energy, and selected nutrients were calculated using an *ad hoc* computer algorithm and a specialised nutritional value calculation software for the BDHQ by EBNJAPAN, a BDHQ service provider. This algorithm incorporates the sex-specific portion size, which is mainly based on recipe books for Japanese dishes,^(15)^ and the nutrient composition of each food item derived from the 2015 version of the Standard Tables of Food Composition in Japan.^(17)^ The intake of dietary supplements was not included in the analysis owing to the absence of a reliable composition table for dietary supplements in Japan.

No self-administered dietary assessment can completely eliminate reporting errors, particularly those related to under- or over-reporting.^(18,19)^ Consequently, the percentage of daily energy intake was calculated using the raw values of total fat, protein, and carbohydrate intake. The intakes of most nutrients were positively correlated with energy intake. Additionally, in the previous validation study of the BDHQ, the significant correlation coefficients of food group and nutrient intakes between the 16-day dietary records and BDHQ were determined when the energy-adjusted values were used.^(15,16)^ Thus, food group and nutritional intake values were adjusted for energy using the density method (amounts per 1000 kcal of energy) to minimise the influence of energy intake and dietary misreporting.^(18,19)^

### Determination of nutritional adequacy

The intake adequacy of each nutrient was assessed using a method reported in previous studies,^(20–23)^ which involved comparing nutrient levels with published dietary reference intakes (DRIs) for the Japanese.^(24)^ The Japanese DRIs provide different types of reference values based on their intended purpose. The tentative dietary goal for preventing lifestyle-related diseases (DG) is to prevent noncommunicable diseases, whereas the estimated average requirement (EAR) is designed to prevent nutrient insufficiency.^(24)^ The following calculation was used to adjust reported nutrient intakes (except for fat and carbohydrates) and compare them against Japanese DRI values:

adjusted nutrient intake = reported intake/reported energy intake × estimated energy requirement (EER).^(20–23)^

For the seven nutrients in the DG (protein, fat, saturated fatty acid (SFA), carbohydrate, total dietary fibre, sodium, and potassium), intake levels falling outside the range of the corresponding reference values (above and/or below) were considered inadequate.

For the 13 nutrients addressed by the EAR (protein, vitamin A, vitamin B_1_, vitamin B_2_, niacin, vitamin B_6_, vitamin B_12_, folate, vitamin C, calcium, magnesium, zinc, and copper), intake levels below the reference value were considered inadequate using the cut-off point method. Three nutrients (molybdenum, selenium, and iodine) were excluded from this analysis, as they could not be calculated using the BDHQ. For iron, the cut-off point method was unsuitable owing to the severely skewed distribution of iron requirements among menstruating women.^(25–27)^ Therefore, a probability of inadequacy >50% for menstruating women whose iron bioavailability was 15% (<9.3 mg/day) was considered inadequate. Reference values for each nutrient are detailed in Supplementary Table 1.

The nutritional inadequacy of each nutrient was quantified as the proportion of participants whose intake fell below the EAR or outside the range of the DG in each group. The overall nutritional inadequacy of the participants was determined by counting the number of nutrients that did not meet the Japanese DRI values.^(21,22)^

### Other variables

In the BDHQ, the participants reported their sex (male or female) and their birth date, body height, and weight. Body mass index (BMI) was calculated as the current body weight (kg) divided by the square of body height (m) and categorised as underweight (less than 18.5 kg/m^2^), normal weight (18.5–25 kg/m^2^), or overweight and obese (25 kg/m^2^ and more).

In the lifestyle questionnaire, participants reported their study year (1 to 4), course (non-health-related/health-related), living status (living alone/living with their family or living in a dormitory), monthly discretionary spending (less than JP¥20,000/JP¥20,000–30,000/JP¥30,000–50,000/JP¥50,000 or more), physical activity score (0–3), and current smoking (yes/no). The physical activity score was determined by tallying affirmative responses to three yes/no questions concerning physical activity, namely, 1) ‘Are you in the habit of doing exercise to sweat lightly for over 30 min at a time, two times weekly, for over a year?’ 2) ‘In your daily life, do you walk or do any equivalent amount of physical activity for more than one hour a day?’ and 3) ‘Is your walking speed faster than the speed of others of your age and sex?’. Higher scores indicated significantly higher levels of physical activity with a metabolic equivalent of a task score of 3 or higher.^(28)^

### Statistical analysis

The differences in characteristics among the low, medium, and high nutrition knowledge groups were assessed using the chi-square test for categorical variables and the Kruskal–Wallis test for continuous variables. For variables that were significant, adjusted *P*-values were calculated using *post-hoc* Bonferroni corrections for multiple comparisons. To compare the characteristics of included and excluded participants, the chi-square test was conducted for categorical variables whereas the Mann–Whitney *U* test was conducted for continuous variables. The differences in mean dietary intakes and nutritional inadequacy among the three groups were compared using covariate analysis to adjust for sex, year, BMI, physical activity score, living status, and monthly discretionary spending as confounding variables that exhibited significant differences (*P* < 0.05) between groups categorised by nutrition knowledge. The nutritional inadequacy of the participants was determined by counting the number of nutrients that did not meet the Japanese DRI values; this included 14 nutrients with EAR and 5 with DG. Furthermore, multiple regression analysis was performed to examine the dose-response relationship of nutrition knowledge level with dietary intakes and nutritional inadequacy. The dependent variables were dietary intakes and nutritional inadequacy, and the explanatory variables were nutritional knowledge level, sex, year, BMI, physical activity score, living status, and monthly discretionary spending. The *P*-values for the linear trend of dietary intakes and prevalence of nutritional inadequacy between the groups were estimated by modelling the frequency categories as a continuous variable. In addition to the significance probability of nutrition knowledge level, the significance probability was calculated for the linear regression model. Logistic regression analysis adjusted for confounders was used to examine differences in the prevalence of not meeting the Japanese DRIs between the low and medium or high groups based on nutrition knowledge. Moreover, for nutrients with both maximum and minimum reference values (protein, fat, and carbohydrate [%energy]), chi-square tests were performed to compare the respective proportions of participants with nutrient intakes above and below the reference values.

To perform a subgroup analysis, nutrition knowledge was divided into three groups based on majors. Using the above model, an analysis of covariance was conducted on the relationship between the three nutrition knowledge groups by major and the number of nutrients that did not meet the Japanese DRIs.

All statistical analyses were conducted using the IBM SPSS statistics software package (version 29.0; SPSS Inc., Chicago, IL, USA). All reported *P*-values were two-tailed, with a *P*-value ≤ 0.05 considered statistically significant.

## Results

To collect data, questionnaires were distributed to 2,036 eligible students, of which 1,372 were returned (response rate: 67.4%). Of these, 1,295 students agreed to participate (participation rate: 63.6%).

The following participants were excluded: those who did not complete the questionnaire (*n* = 103), those with missing data for variables required for statistical analysis (*n* = 70), those with a reported energy intake of less than half the energy requirement for the lowest physical activity category according to the Japanese DRIs or more than 1.5 times the energy requirement for the highest physical activity category (*n* = 319),^(24,29)^ and those with a BMI deviating by more than two standard deviations (SDs) from the average for Japanese individuals aged 20–29 years (mean ± 2SD: male, 22.10 ± 7.0; female, 21.00 ± 6.8)^(5)^ (*n* = 31). As body height and weight were self-reported, some participants had clearly illogical BMI values. Therefore, a cut-off value was set based on the BMI that was representative of Japanese people, which resulted in the inclusion of a final sample of 801 participants for analysis (39.3%). According to the power analysis conducted using G*Power 3,^(30)^ the number of participants required to detect a middle effect size (f = 0.25) with a significance level of *P* = 0.05, a statistical power of 0.8, and with seven covariates was estimated to be 158.^(23,31)^ Thus, the number of participants in the present study was sufficient to evaluate statistically significant differences.

The participants’ basic characteristics are listed in Table 1. The total NKQ scores (mean ± SD) of the low, medium, and high nutrition knowledge groups were 48.1 ± 13.8%, 68.3 ± 2.8%, and 78.5 ± 4.2%, respectively. Kruskal–Wallis test and *post-hoc* Bonferroni corrections revealed significant differences among the three groups and all pairs (all *P* < 0.001). In the high nutrition knowledge group, the proportion of females was higher than that in the medium nutrition knowledge group (low, 52.5%; medium, 45.2%; and high, 59.5%). In the high nutrition knowledge group, there was a lower prevalence of sophomore (year 2) students and a higher prevalence of junior students (year 3). Moreover, the high nutrition knowledge group encompassed fewer students majoring in non-health-related courses and more students majoring in health-related courses than the low and medium nutrition knowledge groups. Energy intake was significantly lower in the high nutrition knowledge group than in the medium nutrition knowledge group, whereas the physical activity score was significantly higher in the medium nutrition knowledge group than in the low and high nutrition knowledge groups (*P* < 0.01). Age, living status, monthly discretionary spending, BMI, and current smoking status did not significantly differ among the three nutrition knowledge groups. Compared with the excluded participants, the included participant group exhibited significantly higher NKQ scores, higher proportions of women and people living alone, and significantly lower numbers of nutrients that did not meet the Japanese DRIs (Supplementary Table 2). Furthermore, the distribution of grade, monthly discretionary spending, and body type differed significantly.

**Table 1.** Characteristics of study participants categorised into high, medium, and low groups based on the nutrition knowledge level

All (*n* = 801)			Low (*n* = 276)		Medium (*n* = 261)		High (*n* = 264)		*P*-value
NKQ score, Mean, SD	64.7	15.4	48.1^a^	13.8	68.30^b^	2.8	78.5^c^	4.2	<0.001
Sex, *n*, (%)									0.005
Men	381	(47.6)	131^a,b^	(47.5)	143^b^	(54.8)	107^a^	(40.5)	
Women	420	(52.4)	145^a,b^	(52.5)	118^b^	(45.2)	157^a^	(59.5)	
Age (years), Mean, SD	19.5	1.2	19.5	1.2	19.5	1.2	19.6	1.3	0.266
Grade, *n*, (%)									<0.001
Freshman (1st year)	404	(50.4)	139^a^	(50.4)	139^a^	(53.3)	126^a^	(47.7)	
Sophomore (2nd year)	146	(18.2)	63^a^	(22.8)	52^a^	(19.9)	31^b^	(11.7)	
Junior (3rd year)	199	(24.8)	55^a^	(19.9)	56^a^	(21.5)	88^b^	(33.3)	
Senior (4th year)	52	(6.5)	19^a^	(6.9)	14^a^	(5.4)	19^a^	(7.2)	
Course, *n*, (%)									<0.001
Non-health	377	(47.1)	164^a^	(59.4)	131^a^	(50.2)	82^b^	(31.1)	
Health	424	(52.9)	112^a^	(40.6)	130^a^	(49.8)	182^b^	(68.9)	
Living status, *n*, (%)									0.425
Living alone	190	(23.7)	58	(21.0)	66	(25.3)	66	(25.0)	
Living with their family or living in a dormitory	611	(76.3)	218	(79.0)	195	(74.7)	198	(75.0)	
Monthly discretionary spending, *n*, (%)									0.059
Less than JP¥20,000	261	(32.6)	89	(32.2)	87	(33.3)	85	(32.2)	
JP¥20,000–30,000	157	(19.6)	58	(21.0)	52	(19.9)	47	(17.8)	
JP¥30,000–50,000	213	(26.6)	77	(27.9)	53	(20.3)	83	(31.4)	
JP¥50,000 or more	170	(21.2)	52	(18.8)	69	(26.4)	49.0	(18.6)	
Body mass index (kg/m^2^), Mean, SD	20.9	2.4	20.8	2.3	20.9	2.4	21.0	2.6	0.647
Body mass index, *n*, (%)									0.379
Underweight (less than 18.5 kg/m^2^)	116	(14.5)	39	(14.1)	36	(13.8)	41	(15.5)	
Normal weight (18.5–25 kg/m^2^)	633	(79.0)	225	(81.5)	207	(79.3)	201	(76.1)	
Overweight and obese (25 kg/m^2^ and more)	52	(6.5)	12	(4.3)	18	(6.9)	22	(8.3)	
Energy intake (kcal/day), Mean, SD	1852	686.0	1874^a,b^	725.0	1938^a^	734.0	1744^b^	576.0	0.027
Current smoking, *n*, (%)									0.108
Yes	62	(7.7)	26	(9.4)	23	(8.8)	13	(4.9)	
No	739	(92.3)	250	(90.6)	238	(91.2)	251	(95.1)	
Physical activity score (0–3), Mean, SD	1.4	1.0	1.3^a^	1.0	1.7^b^	0.9	1.3^a^	1.0	<0.001

NKQ, nutrition knowledge questionnaire; SD, standard deviation.

*P*-values were calculated using the chi-square test for categorical variables and the Kruskal–Wallis test for continuous variables between the low, medium, and high nutrition knowledge groups. Adjusted *P*-values were calculated using *post-hoc* Bonferroni corrections. Identical superscript lowercase letters represent no significant difference between groups.

The habitual daily nutrient intake and inadequate intake prevalence of each nutrient among the 801 university students are shown in Table 2. Vitamin A intake was significantly higher in the high nutrition knowledge group than in the medium nutrition knowledge group (*P* = 0.037). The daily intake of most nutrients tended to be higher in the high nutrition knowledge group than in the low nutrition knowledge group, although the differences were not statistically significant. A higher knowledge level was significantly associated with higher folate (*P*-trend = 0.035) and zinc (*P*-trend = 0.032) intakes. Both linear regression models were demonstrated to be appropriate with significance levels of *P* < 0.001. The proportion of students with inadequate vitamin B_2_ and folate intake was significantly lower in the high nutrition knowledge groups than in the low nutrition knowledge group. Details of the number of participants above or below the recommendations for each nutrient intake group classified as inadequate are shown in Supplementary Table 3. No significant differences were observed in the proportion of participants above or below the reference values for any of the nutrients (protein, fat, and carbohydrate).

**Table 2.** Habitual daily nutrient intake and prevalence of not meeting the estimated average requirement (EAR) or tentative dietary goal (DG) of dietary reference intakes (DRIs) for the Japanese population among 801 university students categorised into low, medium, and high groups based on the nutrition knowledge level

	All (*n* = 801)		Prevalence of inadequacy* (%)	Low (*n* = 276)		Prevalence of inadequacy* (%)	OR	Medium (*n* = 261)		Prevalence of inadequacy* (%)	OR (95%CI)^¶^	High (*n* = 264)		Prevalence of inadequacy* (%)	OR (95%CI)^¶^	*P*-value^§^	*P*-trend^||^
Nutrient intake (unit/1000 kcal or %energy)	Mean	SD		Mean	SD			Mean	SD			Mean	SD				
Nutrient with EAR																	
Protein (g)	37.0	9.0	0.5	37.0	10.0	0.7	1	37.0	8.0	0.4	0.59 (0.05–7.13)	38.0	8.0	0.4	0.43 (0.04–5.38)	0.334	0.18
Vitamin A (µgRAE)^†^	364.0	314.0	29.1	364.0	339.0	32.2	1	335.0^§^	193.0	29.1	0.78 (0.53–1.16)	394.0^§^	376.0	25.8	0.73 (0.49–1.08)	0.037	0.186
Vitamin B1 (mg)	0.40	0.10	71.9	0.40	0.10	72.5	1	0.40	0.10	75.1	1.17 (0.79–1.74)	0.41	0.10	68.2	0.78 (0.53–1.15)	0.453	0.245
Vitamin B2 (mg)	0.7	0.2	16.9	0.7	0.2	22.5	1	0.7	0.2	14.6	0.51 (0.32–0.81)	0.7	0.2	13.3	0.48 (0.30–0.77)	0.160	0.074
Niacin (mgNE)^‡^	14.5	4.3	0.0	14.2	4.7	0.0	–	14.4	3.8	0.0	–	14.8	4.2	0.0	–	0.282	0.109
Vitamin B6 (mg)	0.62	0.19	21.1	0.60	0.20	24.6	1	0.61	0.18	18.4	0.65 (0.42–1.00)	0.63	0.19	20.1	0.73 (0.48–1.13)	0.162	0.058
Vitamin B12 (mg)	4.6	3.1.0	1.9	4.6	3.6.0	2.9	1	4.4	2.4	1.5	0.47 (0.13–1.64)	4.8	3.0	1.1	0.37 (0.09–1.48)	0.295	0.407
Folate (µg)	158.0	68.0	10.2	152.0	67.0	13.4	1	158.0	68.0	10.0	0.73 (0.42–1.26)	164.0	68.0	7.2	0.41 (0.22–0.75)	0.096	0.035
Vitamin C (mg)	52.0	27.0	31.0	52.0	29.0	31.2	1	52.0	29.0	31.4	1.03 (0.71–1.49)	52.0	25.0	30.3	0.95 (0.65–1.38)	0.854	0.698
Calcium (mg)	266.0	110	54.7	265.0	120.0	56.5	1	264.0	109.0	54.0	0.92 (0.65–1.31)	270.0	98.0	53.4	0.86 (0.61–1.23)	0.623	0.469
Magnesium (mg)	118.0	31	44.7	116.0	31.0	46.4	1	117.0	31.0	47.5	1.07 (0.75–1.52)	120.0	31.0	40.2	0.74 (0.52–1.06)	0.256	0.135
Iron (mg)	3.9	1.1	42.7	3.8	1.2	38.8	1	3.9	1.1	31.4	0.83 (0.52–1.33)	4.0	1.2	37.9	0.84 (0.53–1.34)	0.122	0.055
Zinc (mg)	4.4	0.8	6.5	4.4	0.8	7.2	1	4.4	0.8	6.1	0.80 (0.40–1.62)	4.5	0.8	6.1	0.78 (0.39–1.58)	0.074	0.032
Copper (mg)	0.58	0.11	0.5	0.58	0.10	0.7	1	0.57	0.11	0.4	0.56 (0.04–7.00)	0.59	0.12	0.4	0.49 (0.04–6.04)	0.067	0.069
Nutrient with DG																	
Protein (%energy)	14.9	3.5	36.5	14.8	3.8	38.0	1	14.8	3.1	33.3	0.73 (0.51–1.06)	15.1	3.4	37.9	1.00 (0.70–1.44)	0.334	0.18
Fat (%energy)	28.5	6.6	48.8	27.9	6.7	49.3	1	28.8	6.7	49.4	1.06 (0.75–1.49)	28.8	6.5	47.7	0.94 (0.67–1.33)	0.051	0.105
SFA (%energy)	8.1	2.3	67.3	8.0	2.5	63.4	1	8.2	2.4	68.2	1.43 (0.98–2.08)	8.1	2.1	70.5	1.41 (0.97–2.05)	0.117	0.495
Carbohydrate (%energy)	53.5	9.1	37.8	54.1	9.4	39.5	1	53.5	8.7	34.5	0.81 (0.57–1.16)	52.9	9.2	39.4	1.02 (0.71–1.44)	0.233	0.127
Total dietary fibre (g)	5.5	2.0	94.9	5.4	1.9	95.7	1	5.5	2.1	94.3	0.84 (0.38–1.86)	5.7	1.8	94.7	0.81 (0.36–1.81)	0.296	0.13
Sodium (salt-equivalent) (g)	5.7	1.2	98.8	5.8	1.3	97.5	–	5.7	1.2	100.0	–	5.6	1.2	100.0	–	0.211	0.084
Potassium (mg)	1188.0	388.0	57.9	1167.0	381.0	61.2	1	1182.0	402.0	57.5	0.89 (0.62–1.27)	1214.0	380.0	54.9	0.72 (0.50–1.03)	0.323	0.15

OR, odds ratio; SD, standard deviation; 95% CI, 95% confidence interval; RAE, retinol activity equivalent.

*The percentage of participants whose nutrient intake did not meet the DG or EAR of Japanese DRIs is shown. Adjustment of the reporting error was performed using the following formula: Nutrient intake = reported nutrient intake/reported energy intake × EER. The EER of physical activity level II for 18–29-year-old Japanese males and females is 2650 kcal/day and 2000 kcal/day, respectively.^(13)^ Each nutrient intake was compared with each Japanese DRI value using the cut-point method.

^†^Sum of retinol, β-carotene/12, α-carotene/24, and cryptoxanthin/24.

^‡^Sum of niacin and protein/6000.

^§^The *P*-values are shown for covariate analysis for differences in nutrient intake between the low, medium, and high nutrition knowledge groups adjusted for confounding variables of sex, study year, body mass index, physical activity score, living status, and monthly discretionary spending.

^||^Multiple regression analysis was used to test the association between the low, medium, and high nutrition knowledge groups and nutrient intakes. Each knowledge group was assigned a score: low = 1, medium = 2, and high = 3. The dependent variables were dietary intakes and nutritional inadequacy, and the explanatory variables were nutritional knowledge level, sex, year, BMI, physical activity score, living status, and monthly discretionary spending.

^¶^Multivariate adjusted ORs for nutrient intake inadequacy between the high and low groups were calculated by adjusting for sex, study year, body mass index, physical activity score, living status, and monthly discretionary spending.

Table 3 shows the overall nutritional inadequacy of 801 university students. The total number of nutrients not meeting the EAR in the high nutrition knowledge group was lower than that in the low nutrition knowledge group, whereas the total number of nutrients not meeting the DG did not significantly differ among the three groups. Furthermore, higher nutrition knowledge levels were associated with fewer nutrients that did not meet the EAR (*P*-trend = 0.016, linear regression model *P* < 0.001).

**Table 3.** Number of nutrients not meeting the tentative dietary goal (DG) or estimated average requirement (EAR) among 801 university students categorised into low, medium, and high groups based on the nutrition knowledge level

	All (*n* = 801)		Low (*n* = 276)		Medium (*n* = 261)		High (*n* = 264)		*P*-value*	*P*-trend^†^
	Mean	SD	Mean	SD	Mean	SD	Mean	SD		
Nutrients with EAR (0–14)	3.3	2.8	3.6	2.9	3.2	2.6	3.1	2.7	0.046	0.016
Nutrients with DG (0–7)	4.4	1.2	4.4	1.2	4.4	1.2	4.5	1.3	0.844	0.856

SD, standard deviation.

*P-values are shown for covariate analysis for differences between the low, medium, and high nutrition knowledge groups adjusted for confounding variables of sex, study year (1–4), body mass index (underweight, normal weight, or overweight and obese), physical activity score (0–3), living status (living alone, or living with their family or living in a dormitory), and monthly discretionary spending (less than JP¥20,000, JP¥20,000–30,000, JP¥30,000–50,000, or JP¥50,000 or more).

^†^Multiple regression analysis was used to test the trend of association between the low, medium, and high nutrition knowledge groups and number of nutrients with inadequate intake. Each knowledge group was assigned a score: low = 1, medium = 2, and high = 3. The dependent variables were dietary intakes and nutritional inadequacy, and the explanatory variables were nutritional knowledge level, sex, year, BMI, physical activity score, living status, and monthly discretionary spending.

The habitual food group intakes of university students are shown in Table 4. The daily noodle intake was significantly lower in the high nutrition knowledge group than in the low nutrition knowledge group, whereas potato intake was significantly higher in the high nutrition knowledge group than in the low nutrition knowledge group. Additionally, the daily intake of fruit and vegetable juice was significantly higher in the low nutrition knowledge group than in the medium and high nutrition knowledge groups, whereas mushroom intake was significantly higher in the high nutrition knowledge group than in the low and medium nutrition knowledge groups. A higher knowledge level was significantly associated with a high intake of fat, oil, fruits, green and yellow vegetables, and mushrooms. The results of statistical analysis by majors of the participants are shown in Supplementary Tables 4–5. Mean nutrition knowledge scores were significantly different across all majors. Additionally, nutrition knowledge scores were the highest among nutrition majors and lowest among non-health-related majors. Analysis by major showed no significant association between the total number of nutrients not meeting the EAR and DG and nutrition knowledge.

**Table 4.** Habitual daily food group intake among 801 university students categorised into low, medium, and high groups based on the nutrition knowledge level*

	All (*n* = 801)		Low (*n* = 276)		Medium (*n* = 261)		High (*n* = 264)		*P*-value^†^	*P*-trend^‡^
Food group intake (g/1000 kcal)	Mean	SD	Mean	SD	Mean	SD	Mean	SD		
Cereals	228.5	81.2	229.1	83.1	227.3	78.6	228.9	82.0	0.640	0.969
Rice	160.1	80.1	155.8	80.4	159.3	79.8	165.2	80.1	0.294	0.155
Bread	21.2	16.8	21.9	18.4	21.7	16.7	20.0	15.1	0.267	0.206
Noodles	47.2	35.4	51.5	39.6	46.3	34.7	43.7	30.6	0.025	0.011
Pulses	26.6	24.2	25.3	22.7	25.1	22.7	29.6	26.9	0.080	0.066
Potatoes	20.8	19.8	18.8	18.6	20.7	20.2	22.9	20.3	0.047	0.014
Sugar	2.2	2.1	2.1	2.2	2.2	2.1	2.3	2.0	0.453	0.244
Confectioneries	24.6	19.5	25.3	19.5	25.2	20.4	23.4	18.7	0.381	0.216
Fat and oil	6.8	3.0	6.5	3.1	6.9	3.0	7.0	3.0	0.072	0.047
Fruits	24.3	28.4	22.0	25.9	26.6	33.4	24.5	25.2	0.101	0.002
Total vegetables	119.6	75.4	116.8	71.7	118.1	78.8	124.1	75.9	0.551	0.311
Green and yellow vegetables	37.4	31.1	34.2	30.4	37.6	31.8	40.6	31.0	0.067	0.022
Other vegetables	59.1	41.0	55.9	39.2	59.7	42.4	61.8	41.4	0.251	0.181
Pickled vegetables	5.5	7.5	6.2	7.8	5.5	7.8	4.7	6.6	0.146	0.047
Mushrooms	4.5	4.7	4.1	4.8	4.1	4.3	5.2	5.0	0.025	0.013
Seaweeds	4.9	5.3	4.8	5.8	4.5	4.7	5.3	5.1	0.211	0.22
Beverages	301.7	228.4	301.1	245.6	318.4	227.8	285.7	209.1	0.275	0.391
Fruit and vegetable juice	36.9	65.7	51.8	92.3	29.3	43.4	29.0	45.0	<0.001	<0.001
Tea	169.9	175.8	154.4	171.2	189.5	186.2	166.9	168.7	0.052	0.496
Coffee	37.3	68.8	31.1	61.7	41.1	76.4	40.0	67.8	0.266	0.171
Soft drinks	57.5	84.8	63.9	93.2	58.5	76.4	49.8	83.1	0.228	0.089
Fish and shellfish	36.1	28.1	36.7	33.7	34.2	22.0	37.4	26.9	0.434	0.673
Meat	47.7	28.7	46.1	28.7	48.9	29.0	48.2	28.2	0.403	0.339
Eggs	24.7	15.5	23.7	15.2	24.4	16.2	26.1	15.1	0.185	0.08
Dairy products	79.6	70.4	81.5	80.6	78.8	69.2	78.4	59.3	0.947	0.717

SD, standard deviation.

*Adjustment of the reporting error was performed using the following formula: Food group intake = reported food group intake/reported energy intake × 1000 (kcal).

^†^The *P*-values are shown for covariate analysis for differences in food group intake between the low, medium, and high nutrition knowledge groups adjusted for confounding variables of sex, study year (1–4), body mass index (underweight, normal weight, or overweight and obese), physical activity score (0–3), living status (living alone, or living with their family or living in a dormitory), and monthly discretionary spending (less than JP¥20,000, JP¥20,000–30,000, JP¥30,000–50,000, or JP¥50,000 or more).

^‡^Multiple regression analysis was used to test the trend of association between the low, medium, and high nutrition knowledge groups and food group intakes. Each knowledge group was assigned a score: low = 1, medium = 2, and high = 3. The dependent variables were dietary intakes and nutritional inadequacy, and the explanatory variables were nutritional knowledge level, sex, year, BMI, physical activity score, living status, and monthly discretionary spending.

## Discussion

In this study, we investigated the association between nutrition knowledge and nutrient adequacy among Japanese university students. Our findings indicate that university students with high nutrition knowledge had higher nutrient intake adequacy than those with low nutrition knowledge. This finding suggests that the nutrition knowledge level affects dietary habits among Japanese young adults. To the best of our knowledge, this study is the first to examine the relationship between nutrition knowledge and overall nutrient intake adequacy among university students in Asia.

The mean score for nutrition knowledge in the present study (64.7%) was similar to that observed in previous studies (approximately 60–70%).^(32–37)^ Moreover, female students had higher mean nutrition knowledge than male students (males, 63.6%; females, 65.7%; *P* = 0.045), which is consistent with the results of several Western and Japanese studies.^(13,32,33,38)^ Notably, research suggests that women tend to consider health- and weight-related motives for food selection more often than men,^(33,39)^ which potentially contributes to a greater preoccupation with food habits among females.

In the present study, no significant relationship was observed between nutrition knowledge and age, which is consistent with the results of some published studies.^(23,32)^ In contrast, some studies revealed a positive correlation between age and nutrition knowledge,^(34,38)^ and some found that middle-aged people had higher nutrition knowledge than older and younger people.^(13,33)^ The age distribution and age categories varied between the study populations in the present and previous studies, and the age range was narrow in the present study (18–27 years). Thus, these observations do not necessarily contradict the findings of the present study.

The higher proportion of students studying health-related courses in the high nutrition knowledge group than in the other groups can be attributed to the inclusion of students majoring in nutrition in this category. Similarly, the association between study year and nutrition knowledge may be explained by the proportion of students majoring in health-related courses. It should be noted that the proportion of students majoring in health-related courses was low in sophomores and high in juniors. Monthly discretionary spending did not differ among the three nutrition knowledge groups. Similarly, in a previous study encompassing Japanese adults, nutrition knowledge was not found to be significantly related to education or household income.^(32)^ In contrast, nutrition knowledge was higher in groups with higher education levels and socioeconomic status among German adults. It is possible that no relationship between nutrition knowledge and socioeconomic variables was observed in the present study because the enrolled participants likely had similar education levels and socioeconomic status. In addition, the lack of association between BMI and nutrition knowledge in the present study was in accordance with the findings of previous studies.^(40,41)^ While a few reports have demonstrated a negative association between nutrition knowledge and BMI, this association was not strong.^(33,34)^ In the present study, there was a higher proportion of participants classified as underweight and normal weight and a lower proportion classified as overweight and obese than those in previous studies. Therefore, this difference in results may be explained by differences in BMI status among the study populations.

We found that university students with high nutrition knowledge exhibited a higher adequacy of nutrient intakes than those with low nutrition knowledge; the group with higher nutrition knowledge exhibited healthier dietary habits. In the present study, higher knowledge levels were significantly associated with higher intakes of potatoes, green and yellow vegetables, and mushrooms. Previous studies have reported an association between dietary patterns and a higher intake of these foods with a lower prevalence of essential micronutrient deficiencies.^(20,42)^ Therefore, the group with high nutrition knowledge in the present study may have a dietary pattern with a lower prevalence of inadequate essential micronutrient intake. Consistent with our findings, several previous studies have also reported a positive correlation between nutrition knowledge and overall diet quality.^(12,43)^ However, previous studies primarily assessed diet quality based on food intake quantity, which differs from the approach employed in our study. Thus, the results of the present study add to those of recent studies, suggesting that nutrition knowledge is an important target for health education and holds the potential to contribute to improving dietary quality.

On the other hand, analysis by major showed no significant association between the total number of nutrients not meeting the EAR and DG and nutrition knowledge. This result may be driven by the narrow distribution of nutrition knowledge scores. Considering that the association became significant as the distribution of nutrition knowledge increased, cursory nutrition education may not be adequate to change eating behaviour; long-term and systematic nutrition education and advanced nutrition knowledge may be necessary to implement such a change. Compared to the excluded participants, the participants included in this study were likely to have higher health literacy, as demonstrated by higher NKQ scores, higher proportion of women, and fewer smokers. Therefore, it is unclear whether similar results would be obtained in groups with particularly low health literacy.

However, caution should be exercised when interpreting the results of this study. Although a correlation was observed between nutrition knowledge and the number of nutrients that did not meet the EAR, no association was identified with the number of nutrients that did not meet the DG. Furthermore, the average number of nutrients not meeting the DG among the participants in the present study was relatively high, with a value of 4.4 (range: 0–7). In this study, the proportion of young adults with inadequate total dietary fibre and excessive sodium intake was particularly high. These results are consistent with those of previous studies in young Japanese adults.^(20,21)^ High sodium intake is a major public health concern in Japan.^(44)^ Additionally, low dietary fibre intake is considered a risk factor for noncommunicable diseases.^(45,46)^ Moreover, the association between nutrition knowledge and dietary intake was limited. Therefore, when implementing interventions aimed at preventing lifestyle-related diseases and improving eating habits among healthy young adults, enhancing nutrition knowledge alone may not be sufficient and may need to be combined with improving other health literacy parameters, such as health risk perception. Longitudinal studies involving understudied populations with low health literacy are needed to better understand the causal and dose-response relationships between nutrition knowledge and dietary habits.

A strength of our study is that we used a validated questionnaire to measure nutrition knowledge. The NKQs for adults used in this study were developed based on several previously validated questionnaires, and the questions and wording were carefully selected after a pilot study.^(11)^ Cronbach’s alpha, after excluding the section on awareness of dietary recommendations, demonstrated internal consistency between the sections, with a score of 0.76. Typically, Cronbach’s alpha >0.7 is considered to indicate sufficient internal consistency.^(47)^ Therefore, the internal consistency of the questionnaires was considered reasonable.

However, this study has several limitations. First, the cross-sectional nature of this study did not permit the assessment of causality or its direction owing to the uncertain temporality of the association. Second, the selection of participants from a university in Hyogo Prefecture, Japan, was not a random sample selection from the general population. Accordingly, the participants are unlikely to be representative of all Japanese young adults. The generalisability of our results should be carefully considered, as commonly consumed foods and food availability differ between areas and between countries. Nevertheless, because associations were observed in a population with a narrow age and geographic distribution and the results were similar to those of previous studies, it is possible that more detailed associations may be observed in populations with broader distributions. Third, the final participation rate was only 39.3%, and the influence of selection bias cannot be denied. In fact, compared to the excluded participants, included participants were likely to have higher health literacy. Additionally, the participants of this study included nutrition majors. Thus, the participants might have been highly health conscious, with a higher nutrition knowledge level than most Japanese people. Although the study participants were likely interested in nutrition and food, the NKQ scores between the three groups categorised by nutrition knowledge level were significantly different. The association between nutrition knowledge level and dietary intake may be more pronounced if we include populations with low health literacy. Further, we conducted a subgroup analysis comparing nutrition majors, other health-related majors, and non-health-related majors separately to provide more nuanced insights into the results. It cannot be denied that the inclusion of nutritional majors may have contributed to the significant association of nutrition knowledge with dietary habits. Nevertheless, our results may explain some of the subtle findings in previous studies, such as the possibility that improving eating habits may require advanced nutritional knowledge. Fourth, as we used the BDHQ to assess dietary intake, the dietary intake could not be measured without error. In fact, 319 participants were excluded from this study because their energy intake was outside the reference range. Fifth, as the body weight and body height values were self-reported, the influence of declaration errors cannot be denied. Lastly, the NKQ for adults was validated in a group with target characteristics different from those in this study, although the internal consistency of the questionnaire was sufficiently high. Thus, future longitudinal studies should aim to comprehensively investigate the relationship between nutrition knowledge and dietary habits, thus expanding the survey area.

In conclusion, the results of this study indicate that the habitual nutrient intake adequacy of Japanese university students is associated with their nutrition knowledge level. These associations observed in Japanese young adults were generally consistent with observations made in Western countries. However, the association between nutrition knowledge and dietary intake was limited and weak, suggesting that the role of nutrition knowledge in improving dietary habits in healthy young adults may be partial. The results of this study provide a reference for subsequent nutritional and health education for young adults. Future longitudinal studies that include understudied populations with low health literacy are needed to clarify the causal and dose-response relationships between nutrition knowledge and dietary habits.

## Supporting information

Yanagihara and Narumi-Hyakutake supplementary material Yanagihara and Narumi-Hyakutake supplementary material

## Supplementary material

The supplementary material for this article can be found at https://doi.org/10.1017/jns.2025.5
