# Supplementary material for: Relationship between nutrition knowledge and nutritional adequacy in Japanese university students: a cross-sectional study
Source: J Nutr Sci. 2025 Feb 5;14:e14. doi: 10.1017/jns.2025.5 (PMC11811863; doi:10.1017/jns.2025.5)
Supplement: Yanagihara and Narumi-Hyakutake supplementary material [file S2048679025000059sup001.docx]

**Supplementary Material**

**Supplementary Table 1.** **Reference values for each nutrient^*^**

|  | **Males** | | **Females** | |
| --- | --- | --- | --- | --- |
| Energy with EER (kcal)^†^ | 2650 | | 2000 | |
|  | **Minimum RV** | **Maximum RV** | **Minimum RV** | **Maximum RV** |
| Nutrient with EAR^‡^ |  |  |  |  |
| Protein (g) | 50 |  | 40 |  |
| Vitamin A (µgRAE) | 600 |  | 450 |  |
| Vitamin B1 (mg) | 1.2 |  | 0.9 |  |
| Vitamin B2 (mg) | 1.3 |  | 1.0 |  |
| Niacin (mgNE) | 13 |  | 9 |  |
| Vitamin B6 (mg) | 1.1 |  | 1.0 |  |
| Vitamin B12 (mg) | 2.0 |  | 2.0 |  |
| Folate (µg) | 200 |  | 200 |  |
| Vitamin C (mg) | 85 |  | 85 |  |
| Calcium (mg) | 650 |  | 550 |  |
| Magnesium (mg) | 280 |  | 230 |  |
| Iron (mg) | 6.5 |  | 9.3§ |  |
| Zinc (mg) | 9 |  | 7 |  |
| Copper (mg) | 0.7 |  | 0.6 |  |
| Nutrient with DG^‡^ |  |  |  |  |
| Protein (%energy) | 13 | 20 | 13 | 20 |
| Fat (%energy) | 20 | 30 | 20 | 30 |
| SFA (%energy) |  | 7 |  | 7 |
| Carbohydrate (%energy) | 50 | 65 | 50 | 65 |
| Total dietary fibre (g) | 21 |  | 18 |  |
| Sodium (salt-equivalent) (g) |  | 7.5 |  | 6.5 |
| Potassium (mg) | 3000 |  | 2600 |  |

EER, estimated energy requirement; DG, dietary goal; EAR, estimated average requirement; NE, niacin equivalent, RAE, retinol activity equivalent; SFA, saturated fatty acid; RV, reference value.

^*^ Dietary reference intakes (DRIs) for Japanese males and females aged 18–29 years.

^†^ Estimated energy requirement for physical activity level Ⅱ for 18–29-year-old Japanese males and females.

^‡^ Nutritional inadequacy was identified if the intake of a nutrient was below the EAR or outside the range of the DG.

§ A probability of inadequacy >50% for menstruating women whose iron bioavailability was 15% (<9.3 mg/day) was considered inadequate.

**Supplementary Table 2. Comparison of the characteristics of included and excluded participants**

|  | **All**  **(*n* =1192)** | | **Included participants**  **(*n* = 801)** | | **Excluded participants**  **(*n* = 391)** | | ***P*-value** |  |
| --- | --- | --- | --- | --- | --- | --- | --- | --- |
| NKQ score, Mean, SD | 61.9 | 17.8 | 64.7 | 15.4 | 56.2 | 20.8 | <0.001 | |
| Sex, *n*, (%) |  |  |  |  |  |  | <0.001 | |
| Men | 617 | (51.8) | 381 | (47.6) | 236 | (60.4) |  | |
| Women | 575 | (48.2) | 420 | (52.4) | 155 | (39.6) |  | |
| Age (years), Mean, SD | 19.6 | 1.2 | 19.5 | 1.2 | 19.7 | 1.2 | 0.022 | |
| Grade, *n*, (%) |  |  |  |  |  |  | 0.008 | |
| Freshman (1st year) | 568  236  296  92 | (47.7) | 404 | (50.4) | 164 | (41.9) |  | |
| Sophomore (2nd year) | 236 | (19.8) | 146 | (18.2) | 90 | (23.0) |  | |
| Junior (3rd year) | 296 | (24.8) | 199 | (24.8) | 97 | (24.8) |  | |
| Senior (4th year) | 92 | (7.7) | 52 | (6.5) | 40 | (10.2) |  | |
| Course, *n*, (%) |  |  |  |  |  |  | <0.001 | |
| Non-health | 605 | (50.8) | 377 | (47.1) | 228 | (58.3) |  | |
| Health | 587 | (49.2) | 424 | (52.9) | 163 | (41.7) |  | |
| Living status, *n*, (%) |  |  |  |  |  |  | <0.001 | |
| Living alone | 325 | (27.3) | 190 | (23.7) | 135 | (34.6) |  | |
| Living with their family or living in a dormitory | 866 | (72.7) | 611 | (76.3) | 255 | (65.4) |  | |
| Monthly discretionary spending, *n*, (%) |  |  |  |  |  |  | <0.001 | |
| Less than JP¥20,000 | 368 | (30.9) | 261 | (32.6) | 107 | (27.4) |  | |
| JP¥20,000–30,000 | 214 | (18.0) | 157 | (19.6) | 57 | (14.6) |  | |
| JP¥30,000–50,000 | 303 | (25.4) | 213 | (26.6) | 90 | (23.0) |  | |
| JP¥50,000 or more | 307 | (25.8) | 170 | (21.2) | 137 | (35.0) |  | |
| Body mass index (kg/m^2^), Mean, SD | 21.2 | 3.2 | 20.9 | 2.4 | 21.8 | 4.4 | 0.063 | |
| Body mass index, *n*, (%) |  |  |  |  |  |  | 0.012 | |
| Underweight (less than 18.5 kg/m^2^) | 170 | (14.3) | 116 | (14.5) | 54 | (13.8) |  | |
| Normal weight (18.5–25 kg/m^2^) | 925 | (77.6) | 633 | (79.0) | 292 | (74.7) |  | |
| Overweight and obese (25 kg/m^2^ and more) | 97 | (8.1) | 52 | (6.5) | 45 | (11.5) |  | |
| Current smoking, *n*, (%) |  |  |  |  |  |  | 0.022 | |
| Yes | 108 | (9.1) | 62 | (7.7) | 46 | (11.8) |  | |
| No | 1083 | (90.9) | 739 | (92.3) | 344 | (88.2) |  | |
| Physical activity score (0–3), Mean, SD | 1.4 | 0.9 | 1.4 | 1.0 | 1.4 | 0.9 | 0.943 | |

NKQ, nutrition knowledge questionnaire; SD, standard deviation. *P*-values were calculated using the chi-square test for categorical variables and the Mann–Whitney *U* test for continuous variables between the included and excluded participants.

**Supplementary Table 3. Details on the participants who were above/below the recommendations for each nutrient intake group classified as inadequate**

|  | **All** | | **Low** | | **Medium** | | **High** | | ***P*-value** |
| --- | --- | --- | --- | --- | --- | --- | --- | --- | --- |
|  | **(*n* = 801)** | | **(*n* = 276)** | | **(*n* = 261)** | | **(*n* = 264)** | |  |
| Protein (%energy),  *n*, (%) |  |  |  |  |  |  |  |  | 0.409 |
| Below reference range | 241 | (30.1) | 88 | (31.9) | 75 | (28.7) | 78 | (29.5) |  |
| Within reference range | 509 | (63.1) | 171 | (62.0) | 174 | (66.7) | 164 | (62.1) |  |
| Above reference range | 51 | (8.3) | 17 | (6.2) | 12 | (4.6) | 22 | (8.3) |  |
| Fats (%energy),  *n*, (%) |  |  |  |  |  |  |  |  | 0.564 |
| Below reference range | 77 | (9.6) | 33 | (12.0) | 22 | (8.4) | 22 | (8.3) |  |
| Within reference range | 410 | (51.2) | 140 | (50.7) | 132 | (50.6) | 138 | (52.3) |  |
| Above reference range | 314 | (39.2) | 103 | (37.3) | 107 | (41.0) | 104 | (39.4) |  |
| Carbohydrates (%energy), *n*, (%) |  |  |  |  |  |  |  |  | 0.348 |
| Below reference range | 235 | (29.3) | 79 | (28.6) | 72 | (27.6) | 84 | (31.8) |  |
| Within reference range | 498 | (62.2) | 167 | (60.5) | 171 | (65.5) | 160 | (60.6) |  |
| Above reference range | 68 | (8.5) | 30 | (10.9) | 18 | (6.9) | 20 | (7.6) |  |

*P*-values were calculated using the chi-square test.

**Supplementary Table 4. Nutrition knowledge scores for the three nutrition knowledge groups by major**

| **Nutrition knowledge score (%), Mean SD** | **All (n = 801)** | | **Low (n = 276)** | | **Medium (n = 261)** | | **High (n = 264)** | | ***P*** |
| --- | --- | --- | --- | --- | --- | --- | --- | --- | --- |
| All majors | 64.7 | 15.4 | 48.1 | 13.8 | 68.3 | 2.8 | 78.5 | 4.2 | <0.001 |
|  | **All (*n* = 156)** | | **Low (*n* = 55)** | | **Medium (*n* = 50)** | | **High (*n* = 51)** | |  |
| Nutrition majors | 72.5* | 10.6 | 60.8 | 7.5 | 74.5 | 2.1 | 83.1 | 3.5 | <0.001 |
|  | **All (*n* = 268)** | | **Low (*n* = 90)** | | **Medium (*n* = 98)** | | **High (*n* = 80)** | |  |
| Other health-related majors | 66.6* | 13.1 | 52.3 | 11.8 | 69.5 | 2.7 | 79.0 | 3.9 | <0.001 |
|  | **All (*n* = 377)** | | **Low (*n* = 133)** | | **Medium (*n* = 127)** | | **High (*n* = 117)** | |  |
| Non-health-related majors | 60.1* | 16.9 | 41.6 | 14.3 | 65.3 | 3.6 | 75.7 | 3.9 | <0.001 |

SD, standard deviation. *P*-values are shown for the Kruskal–Wallis test for continuous variables between the low, medium, and high nutrition knowledge groups. * Indicates that values were significantly different among all majors (*P*<0.01).

**Supplementary Table 5. Number of nutrients not meeting the tentative dietary goal (DG) and estimated average requirement (EAR) in the three nutrition knowledge groups by major**

|  | **Mean** | **SD** | **Mean** | **SD** | **Mean** | **SD** | **Mean** | **SD** | ***P **** | ***P* trend †** |
| --- | --- | --- | --- | --- | --- | --- | --- | --- | --- | --- |
| **Nutrition majors** | **All (*n* = 156)** | | **Low (*n* = 55)** | | **Medium (*n* = 50)** | | **High (*n* = 51)** | |  |  |
| Nutrient with EAR (0–14) | 3.5 | 2.8 | 4.1 | 2.9 | 3.3 | 2.7 | 3.1 | 2.7 | 0.223 | 0.135 |
| Nutrient with DG (0–7) | 4.4 | 1.3 | 4.4 | 1.3 | 4.4 | 1.2 | 4.4 | 1.4 | 0.842 | 0.559 |
| **Other health-related majors** | **All (*n* = 268)** | | **Low (*n* = 90)** | | **Medium (*n* = 98)** | | **High (*n* = 80)** | |  |  |
| Nutrient with EAR (0–14) | 3.0 | 2.6 | 3.2 | 2.8 | 2.8 | 2.2 | 3.1 | 2.6 | 0.579 | 0.892 |
| Nutrient with DG (0–7) | 4.4 | 1.2 | 4.4 | 1.2 | 4.4 | 1.3 | 4.3 | 1.2 | 0.963 | 0.852 |
| **Non-health-related majors** | **All (*n* = 377)** | | **Low (*n* = 133)** | | **Medium (*n* = 127)** | | **High (*n* = 117)** | |  |  |
| Nutrient with EAR (0–14) | 3.4 | 2.9 | 3.6 | 3.0 | 3.4 | 2.9 | 3.3 | 2.6 | 0.485 | 0.234 |
| Nutrient with DG (0–7) | 4.4 | 1.2 | 4.5 | 1.2 | 4.4 | 1.1 | 4.4 | 1.2 | 0.656 | 0.540 |

SD, standard deviation; * *P*-values are shown for covariate analysis for differences between the low, medium, and high nutrition knowledge groups adjusted for confounding variables of sex, grade (freshman, sophomore, junior, or senior), body mass index (underweight, normal weight, or overweight and obesity), physical activity score (0–3), living status (living alone, or living with their family or living in a dormitory), and monthly discretionary spending (less than JP¥20,000, JP¥20,000–30,000, JP¥30,000–50,000, or JP¥50,000 or more); † Multiple regression analysis was used to test the trend of association between the low, medium, and high nutrition knowledge groups and number of inadequate nutrients. Each knowledge group was assigned a score: Low = 1, Medium = 2, and High = 3. The dependent variables were dietary intakes and nutritional inadequacy, and the explanatory variables were nutritional knowledge level, sex, year, BMI, physical activity score, living status, and monthly discretionary spending.
